# A machine learning model integrating clinical-radiomics-deep learning features accurately predicts postoperative recurrence and metastasis of primary gastrointestinal stromal tumors

**DOI:** 10.1186/s13244-025-02011-8

**Published:** 2025-06-26

**Authors:** WenJie Xie, Zhen Zhang, Zhao Sun, XiaoChen Wan, JieHan Li, JianWu Jiang, Qi Liu, Ge Yang, Yang Fu

**Affiliations:** 1 Department of Gastrointestinal Surgery, The First Affiliated Hospital of Zhengzhou University, Zhengzhou, China; 2 Department of Gastrointestinal Surgery, Henan Cancer Hospital, Zhengzhou, China; 3 Ophthalmology, The First Affiliated Hospital of Zhengzhou University, Zhengzhou, China

**Keywords:** Gastrointestinal stromal tumors, Radiomics, Machine learning, Deep learning, Recurrence or metastasis

## Abstract

**Objectives:**

Post-surgical prediction of recurrence or metastasis for primary gastrointestinal stromal tumors (GISTs) remains challenging. We aim to develop individualized clinical follow-up strategies for primary GIST patients, such as shortening follow-up time or extending drug administration based on the clinical deep learning radiomics model (CDLRM).

**Methods:**

The clinical information on primary GISTs was collected from two independent centers. Postoperative recurrence or metastasis in GIST patients was defined as the endpoint of the study. A total of nine machine learning models were established based on the selected features. The performance of the models was assessed by calculating the area under the curve (AUC). The CDLRM with the best predictive performance was constructed. Decision curve analysis (DCA) and calibration curves were analyzed separately. Ultimately, our model was applied to the high-potential malignant group vs the low-malignant-potential group. The optimal clinical application scenarios of the model were further explored by comparing the DCA performance of the two subgroups.

**Results:**

A total of 526 patients, 260 men and 266 women, with a mean age of 62 years, were enrolled in the study. CDLRM performed excellently with AUC values of 0.999, 0.963, and 0.995 for the training, external validation, and aggregated sets, respectively. The calibration curve indicated that CDLRM was in good agreement between predicted and observed probabilities in the validation cohort. The results of DCA’s performance in different subgroups show that it was more clinically valuable in populations with high malignant potential.

**Conclusion:**

CDLRM could help the development of personalized treatment and improved follow-up of patients with a high probability of recurrence or metastasis in the future.

**Critical relevance statement:**

This model utilizes imaging features extracted from CT scans (including radiomic features and deep features) and clinical data to accurately predict postoperative recurrence and metastasis in patients with primary GISTs, which has a certain auxiliary role in clinical decision-making.

**Key Points:**

- We developed and validated a model to predict recurrence or metastasis in patients taking oral imatinib after GIST.
- We demonstrate that CT image features were associated with recurrence or metastases.
- The model had good predictive performance and clinical benefit.

**Graphical Abstract:**

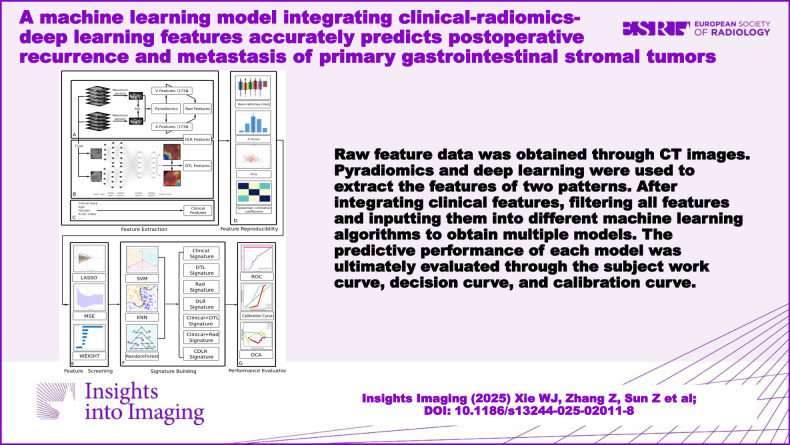

## Introduction

The incidence of gastrointestinal stromal tumors (GISTs) has shown a marked increase over the years, as highlighted by the Surveillance, Epidemiology, and End Results (SEER) database, which reported a rise from 0.55 per 100,000 population in 2001 to 0.78 per 100,000 population in 2011 [1]. Most studies estimate the annual incidence to be approximately 10–15 cases per million individuals [2]. Historically, complete surgical resection (R0 resection) has been the primary treatment for localized and primary GISTs. However, despite its central role, the 5-year survival rate following complete resection remains around 50% [3].

Previous studies have also evaluated surgical resection of metastatic tumors in advanced GIST patients undergoing imatinib therapy. Unfortunately, these trials failed to demonstrate a significant advantage of surgery over the primary endpoint of progression-free survival (PFS) and were terminated prematurely due to the cumulative adverse effects observed [4]. One persistent challenge in the clinical management of GISTs is the accurate prediction of disease recurrence or progression to guide adjuvant therapy decisions. The absence of robust, molecularly defined risk factors leaves many patients untreated, potentially leading to disease recurrence, while others may be unnecessarily overtreated despite a low likelihood of recurrence [5].

Currently, outcomes for patients with advanced GISTs remain suboptimal, with limited evidence to confirm whether surgery improves outcomes in advanced or metastatic disease during the era of targeted therapy [6]. While research is ongoing to improve treatment strategies, randomized trials are lacking, and prognostic outcomes remain poor.

GISTs are known for their genetic heterogeneity, with various mutations influencing their response to imatinib. For instance, studies have shown that most of the survival benefit from imatinib occurs in patients with KIT exon 11 mutations, while those with wild-type GISTs or other KIT or PDGFRA mutations do not derive significant benefits from prolonged adjuvant imatinib therapy [7]. Findings from the Scandinavian Sarcoma Group (SSG) XVIII trial further corroborated that patients with wild-type GISTs or other mutations also fail to benefit from extended imatinib treatment [7].

In clinical practice, the duration of imatinib therapy often varies among individuals, with treatment discontinuation being common due to drug-related adverse effects. Recurrence or metastasis (RM) may occur even in patients with a low mitotic rate, whereas some high-risk patients remain free of RM for extended periods post-surgery. Even with the modified National Institutes of Health (NIH) risk stratification, accurately predicting future RM in GIST patients remains challenging [8].

Recent studies have explored imaging histology-based models for individualized prediction of malignancy in GIST patients. Caiyue Ren et al [9] and Tao Chen et al [10] developed such models to estimate malignancy potential in GISTs, but these tools fall short of directly predicting RM. While non-invasive risk stratification offers clinical value, precision medicine demands tools capable of directly forecasting RM. The prognosis for patients who develop RM remains poor, with significantly reduced overall survival.

Radiomics has emerged as a promising approach for extracting quantitative features from medical images and transforming them into high-dimensional, accessible data [11–15]. This technique analyzes the distribution and relationships of pixel or voxel grayscale values to provide insights into the tumor microenvironment [16]. In this study, we utilized a convolutional neural network (CNN) architecture to construct our model. CNNs process input image data through iterative convolutional and nonlinear operations, transforming raw data into a probability distribution of potential image classes [17].

Our objective is to predict early recurrence and metastasis following GIST surgery, laying the foundation for personalized clinical follow-up strategies and improving patient outcomes.

## Material and methods

### Patients

**Fig. 1:**
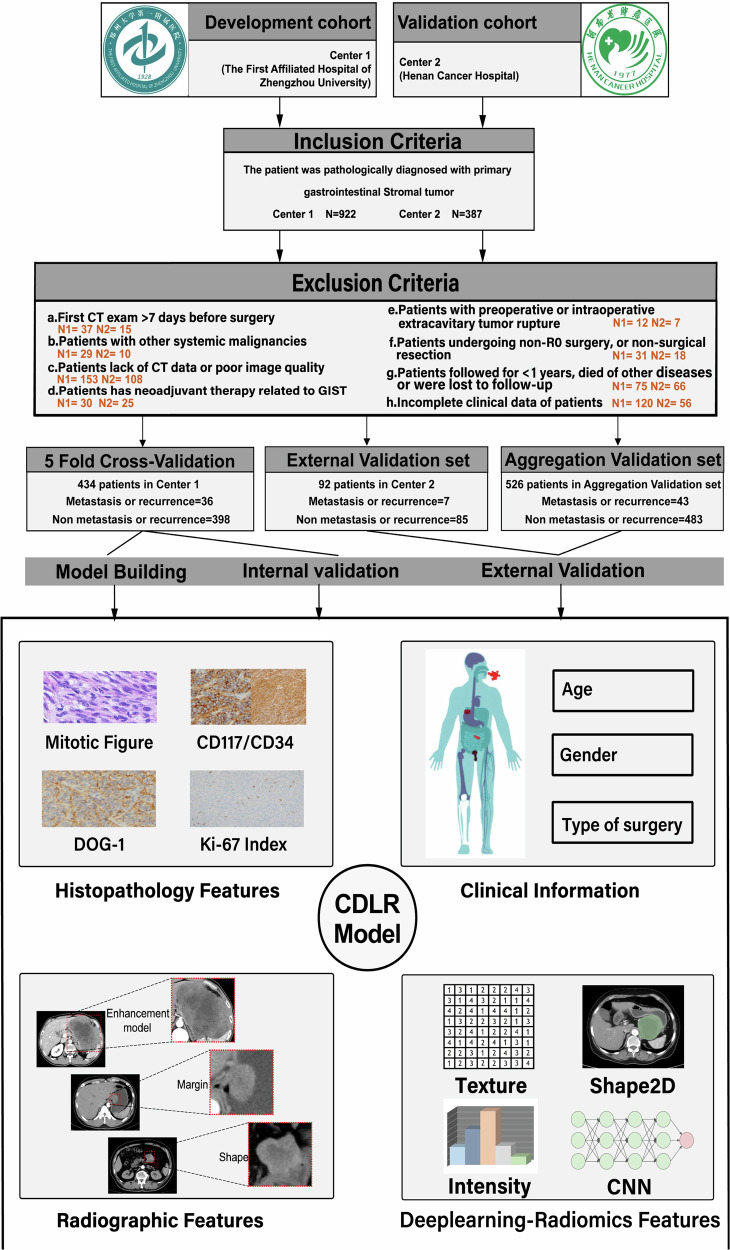
Screening criteria for this study

This retrospective study was approved by the review committee of our institution and was adherent to the principles and requirements of the Declaration of Helsinki. For this retrospective study, we consecutively retrieved patients with pathologically confirmed GISTs from 2015 to 2019 through the picture archiving and communication system of the two hospitals: Affiliated Hospital of Zhengzhou University (center 1) and Henan Cancer Hospital (center 2). This study was approved by the ethics committee of the First Affiliated Hospital of Zhengzhou University, as well as Henan Cancer Hospital, Written informed consent was waived due to the retrospective nature of this study. The patient selection criteria were summarized in Fig. 1. Detailed information on the definitions of the terms related to this article can be found in Supplementary Information 1.

**Fig. 2:**
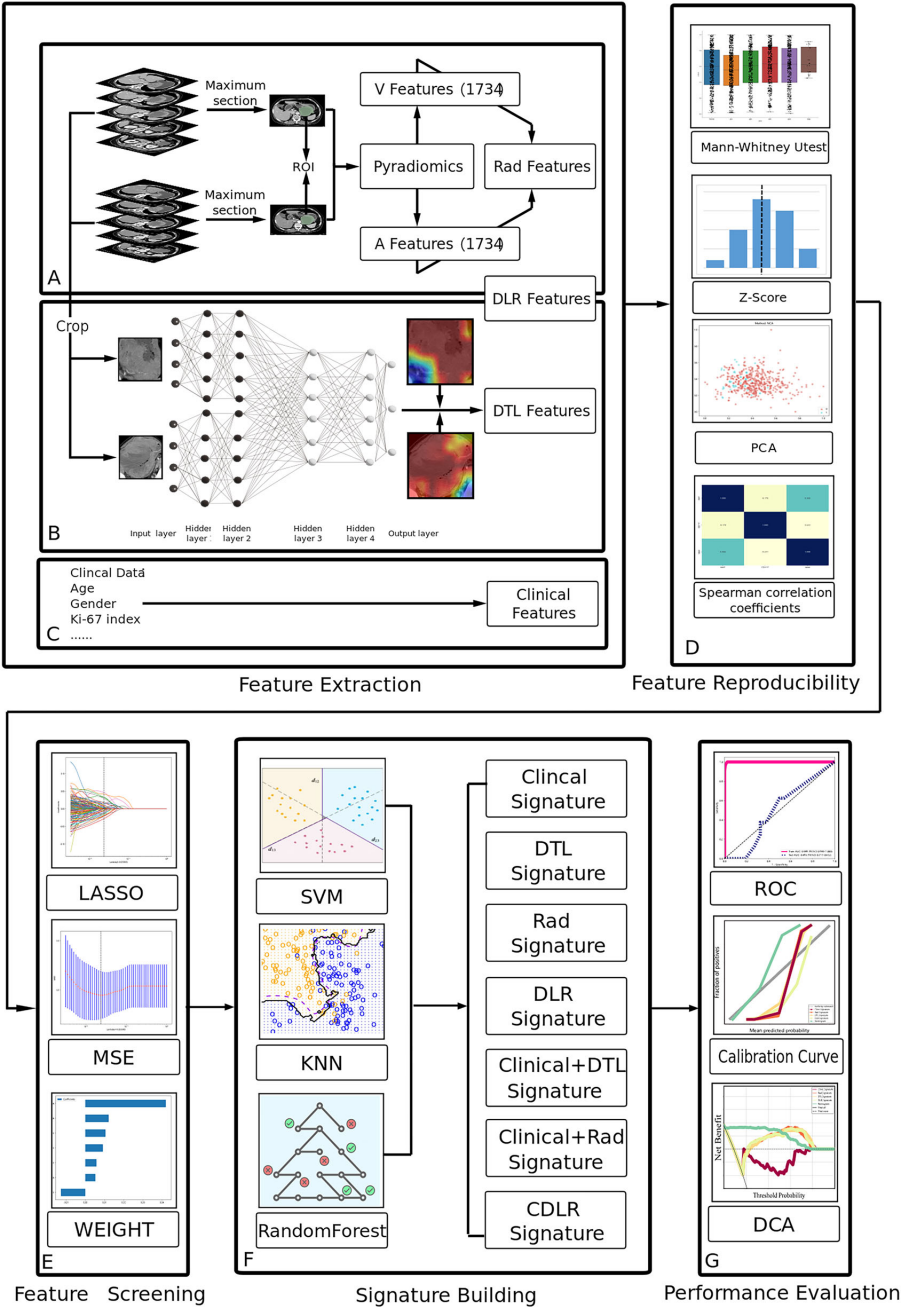
**A**, **B** Extracts radiomics features and deep learning features from the original image data separately; **C** identifying clinical features; **D**, **E** show the selection of highly correlated features for each of the three types of features; **F** inputting the filtered features into multiple machine learning algorithms to establish a model; and **G** evaluating model performance through ROC, DCA, and calibration curves

Based on the inclusion and exclusion criteria, we enrolled a total of 526 GIST patients. Participants were divided into three independent cohorts. The data from center 1, from 2015 to 2019, were divided into a training set and an internal validation set using a five-fold cross-validation method (80% and 20% data split). To create the external validation set, we used data from center 2 for a period between 2016 and 2019. We also aggregated data from the two centers to generate an additional validation set. The workflow was shown in Fig. 2.

### Statistical methods

All statistical analyses were performed using Python (version 3.7.12), and in particular, the statsmodels module (version 0.13.2). Descriptive statistical analyses were performed on clinical data from the training and validation sets by *t*-test or chi-square test. We regarded *p* < 0.05 (two-sided) as statistically significant. We performed imputation for missing values in our study. For all discrete variables, we filled the missing values with the median, and for all continuous variables, we filled the missing values with the mean.

### Feature processing

#### Clinical features

Following National Comprehensive Cancer Network (Version 2.2024) guidelines, we recorded the expression of CD117, CD34, Dog-1, S-100, and Ki-67 indices. We also recorded other clinical information such as age, sex, type of surgery, risk classification, and mitotic index. We acquired CT features from enhanced CT images, using arterial and portal phases from biphasic imaging. Following the Chinese Society of Clinical Oncology guidelines, the CT features extracted are detailed in Supplementary Information 2 [18]. A total of four gastrointestinal surgeons with at least 7 years of experience in abdominal CT reading participated in this study. Three of the doctors were from center 1 and one from center 2. They only know that the patient had been diagnosed with GIST. In this study, the drawing of regions of interest (ROI) in CT images and the determination of CT image features were both dominated by them. All physicians independently reviewed CT images. When there were differences in the judgment results of CT image features, consensus was reached through discussion. The CT protocol was detailed in Supplementary Information 3. In Supplementary Information 4, the detailed process of radiomic features, as well as deep transfer learning (DTL) feature processing, was described.

#### Pre-feature fusion

Features within the unscreened independent feature sets were pre-fused to obtain four fused feature sets: Radiomics + DTL (DLR), Clinical + DTL, Clinical + Radiomics, and Clinical + DTL + Radiomics. The optimal coefficients λ were then found using the Least Absolute Shrinkage and Selection Operator (LASSO) with 10-fold cross-validation. We retained features with non-zero coefficients for linear combination to create rad-scores for these feature sets.

### Model development

We developed the optimal prediction model by combining nine classical machine learning algorithms: linear regression (LR), support vector machine (SVM), K-nearest neighbor, decision tree, random forest, extra-trees, eXtreme Gradient Boosting (XGBoost), light gradient-boosting machine, and multilayer perceptron.

Based on the seven types of feature sets screened from the training set, we trained a total of 63 models by inputting each machine learning algorithm (see above). Due to the small number of recurrent or metastatic samples, the sample imbalance was caused (the distribution of real-world samples was the main reason for this result), and traditional machine learning algorithms might tend to lean towards the dominant categories, leading to a decrease in model performance. To address this issue, we adopted the SMOTE oversampling method, which increases the number of minority class samples in the dataset by synthesizing them to achieve sample balance. This can improve the training effectiveness of the model. Through smote oversampling, the model can better learn features from minority categories, thereby improving its generalization ability and accuracy. In addition, the SMOTE oversampling method can also reduce the overfitting tendency of the model and improve its robustness.

We subsequently validated the results using data from the internal validation set, external validation set, and aggregated set. We then compared prediction results for these data sets across different models. To facilitate comparison, we selected the model with the best external validation for each type of feature, and used the resulting model set for further comparison of calibration curves and decision curve analysis (DCA). We confirmed that CDRLM carried the best application potential.

Clinical deep learning radiomics model (CDLRM) integrates clinical features, radiomic features, and is extracted by DTL. We applied a pre-fusion method to all features, which reduces the number of features lost during modeling and improves model accuracy. To our knowledge, this was the first study to explore whether an integrated model combining deep learning, radiomics, and clinical information can be used as a tool to predict RM. By comparing multiple models, we further investigated whether CDLRM can objectively predict recurrence and metastasis, and attempted to apply the prediction model to clinical decision-making. Details of the algorithms and parameters used for the extraction and modeling of radiomics and deep learning features can be found in Supplementary Information 5.

## Results

### Feature analysis

Tables 1 and 2 list clinical features from GIST patients. In centers 1 and 2, Ki-67, growth pattern, invasion, risk stratification, and mitotic index differed significantly between non-recurrence or metastasis (NRM) and RM groups (*p* < 0.05). However, only in center 1, there were significant differences (*p* < 0.05) in type of surgery, margin contour, tumor shape, CT enhancement mode, intratumoral degeneration, and tumor calcification between the NRM group and RM group, while there was no statistical difference (*p* > 0.05) in center 2. Recurrent patient follow-up information is presented in Supplementary Information 6.

**Table 1:** Clinical information description

	Center 1				Center 2			
	Total	Non-RM	RM	*p*	Total	Non-RM	RM	*p*
Age	58.807 ± 10.429	58.666 ± 10.426	60.361 ± 10.478	0.469	61.130 ± 11.288	61.388 ± 11.259	58.000 ± 12.055	0.338
Gender				0.862				0.178
Male	211 (0.486)*	194 (0.487)	17 (0.472)		49 (0.533)	47 (0.553)	2 (0.286)	
Female	223 (0.514)	204 (0.513)	19 (0.528)		43 (0.467)	38 (0.447)	5 (0.714)	
Max diameter	1.37 ± 1.04	1.37 ± 1.02	1.43 ± 1.26	0.602	1.28 ± 1.20	1.31 ± 1.18	1.00 ± 1.41	0.526
Ki67	9.742 ± 10.567	8.910 ± 9.935	18.944 ± 12.911	< 0.001	11.370 ± 11.704	10.329 ± 10.015	24.000 ± 21.726	0.01
Type of surgery				0.049				0.079
Open surgery	163 (0.376)	144 (0.362)	19 (0.528)		65 (0.707)	58 (0.682)	7 (1.000)	
Laparoscopy surgery	271 (0.624)	254 (0.638)	17 (0.472)		27 (0.294)	27 (0.318)	None	
Margin contour				< 0.001				0.383
Distinct	369 (0.850)	354 (0.889)	15 (0.417)		54 (0.587)	51 (0.600)	3 (0.429)	
Vague	65 (0.150)	44 (0.111)	21 (0.583)		38 (0.413)	34 (0.400)	4 (0.571)	
Tumor shape				< 0.001				0.065
Roundlike	335 (0.772)	322 (0.809)	13 (0.361)		61 (0.663)	59 (0.694)	2 (0.286)	
Lobular	44 (0.101)	32 (0.080)	12 (0.333)		12 (0.130)	9 (0.106)	3 (0.429)	
Irregular	55 (0.127)	44 (0.111)	11 (0.306)		19 (0.207)	17 (0.200)	2 (0.286)	
Growth pattern				0.008				0.003
Internal	170 (0.392)	161 (0.405)	9 (0.250)		37 (0.402)	37 (0.435)	None	
External	159 (0.366)	148 (0.372)	11 (0.306)		40 (0.435)	37 (0.435)	3 (0.429)	
Mixed	105 (0.242)	89 (0.224)	16 (0.444)		15 (0.163)	11 (0.129)	4 (0.571)	
Invasion				< 0.001				0.012
No	316 (0.728)	312 (0.784)	4 (0.108)		42 (0.457)	42 (0.494)	None	
Yes	118 (0.272)	86 (0.216)	32 (0.892)		50 (0.544)	43 (0.506)	7 (1.000)	
CT enhancement mode				< 0.001				0.213
Homogeneity	185 (0.426)	182 (0.457)	3 (0.083)		16 (0.174)	16 (0.188)	None	
Heterogeneity	249 (0.574)	216 (0.543)	33 (0.917)		76 (0.826)	69 (0.812)	7 (1.000)	

* The first number in the table represents the corresponding number of patients, and the last bracket represents the percentage, which is replaced by a decimal number with three decimal places

**Table 2:** Clinical information description

	Center 1				Center 2			
	Total	Non-RM	RM	*p* value	Total	Non-RM	RM	*p* value
Intratumorally degeneration				< 0.001				0.097
No	193 (0.445)	189 (0.475)	4 (0.111)		41 (0.446)	40 (0.471)	1 (0.143)	
Yes	241 (0.555)	209 (0.525)	32 (0.889)		51 (0.554)	45 (0.529)	6 (0.857)	
Tumor calcification				0.018				0.369
No	388 (0.894)	360 (0.905)	28 (0.778)		77 (0.837)	72 (0.847)	5 (0.714)	
Yes	46 (0.106)	38 (0.096)	8 (0.222)		15 (0.163)	13 (0.153)	2 (0.286)	
Risk classification				< 0.001				0.034
Very low	93 (0.214)	89 (0.224)	4 (0.111)		24 (0.261)	23 (0.271)	1 (0.143)	
Low	140 (0.323)	137 (0.344)	3 (0.083)		28 (0.304)	28 (0.329)	None	
Medium	38 (0.088)	38 (0.096)	None		5 (0.054)	5 (0.059)	None	
High	163 (0.376)	134 (0.337)	29 (0.806)		35 (0.380)	29 (0.341)	6 (0.857)	
Mitotic figure				0.027				0.01
(0–5]	90 (0.207)	79 (0.199)	11 (0.306)		13 (0.141)	12 (0.141)	1 (0.143)	
(6–10]	291 (0.671)	282 (0.709)	9 (0.250)		70 (0.761)	68 (0.800)	2 (0.286)	
> 10	53 (0.122)	37 (0.093)	16 (0.444)		9 (0.098)	5 (0.059)	4 (0.571)	
CD34				0.413				0.574
Negative	131 (0.301)	111 (0.279)	7 (0.194)		4 (0.044)	4 (0.047)	None	
Positive	304 (0.699)	287 (0.721)	29 (0.806)		88 (0.957)	81 (0.953)	7 (1.000)	
CD117				0.626				1
Negative	19 (0.044)	18 (0.045)	1 (0.028)		None	None	None	None
Positive	415 (0.956)	380 (0.955)	35 (0.972)		92 (1.000)	85 (1.000)	7 (1.000)	
Dog1				0.538				1
Negative	26 (0.060)	23 (0.058)	3 (0.083)		None	None	None	None
Positive	408 (0.940)	375 (0.942)	33 (0.917)		92 (1.000)	85 (1.000)	7 (1.000)	
S-100				0.836				0.632
Negative	413 (0.952)	379 (0.952)	34 (0.944)		89 (0.967)	82 (0.965)	7 (1.000)	
Positive	21 (0.048)	19 (0.048)	2 (0.056)		3 (0.033)	3 (0.035)	None	

In center 1, the tumors originated from the stomach in 270 patients, while 164 had non-gastric origins, including 121 cases in the small intestine and 43 in other sites. In center 2, 75 patients had gastric origin, 7 had tumors in the duodenum, 16 in the small intestine, and 1 in other sites. Features were extracted from arterial and portal venous phases, encompassing six categories (first order, GLCM, GLDM, GLRM, GLSZM, and shape), resulting in a total of 1734 manual features. These included 360 first-order features, 14 shape features, and a variety of texture features, amounting to 3468 radiomic features when combined across modalities. All manual features were analyzed using an in-house procedure implemented in PyRadiomics. Supplementary Information 7 details all features and their respective *p* values.

The correlation coefficients of DTL features from arterial and portal phases were compared and visualized, revealing weak collinearity between features from different modalities. This finding underscores the capability of deep learning to capture nuanced differences between arterial and portal phase image features. A final set of 64 deep learning features was extracted, with their correlation coefficients presented in Supplementary Information 8.

### Model building

**Fig. 3:**
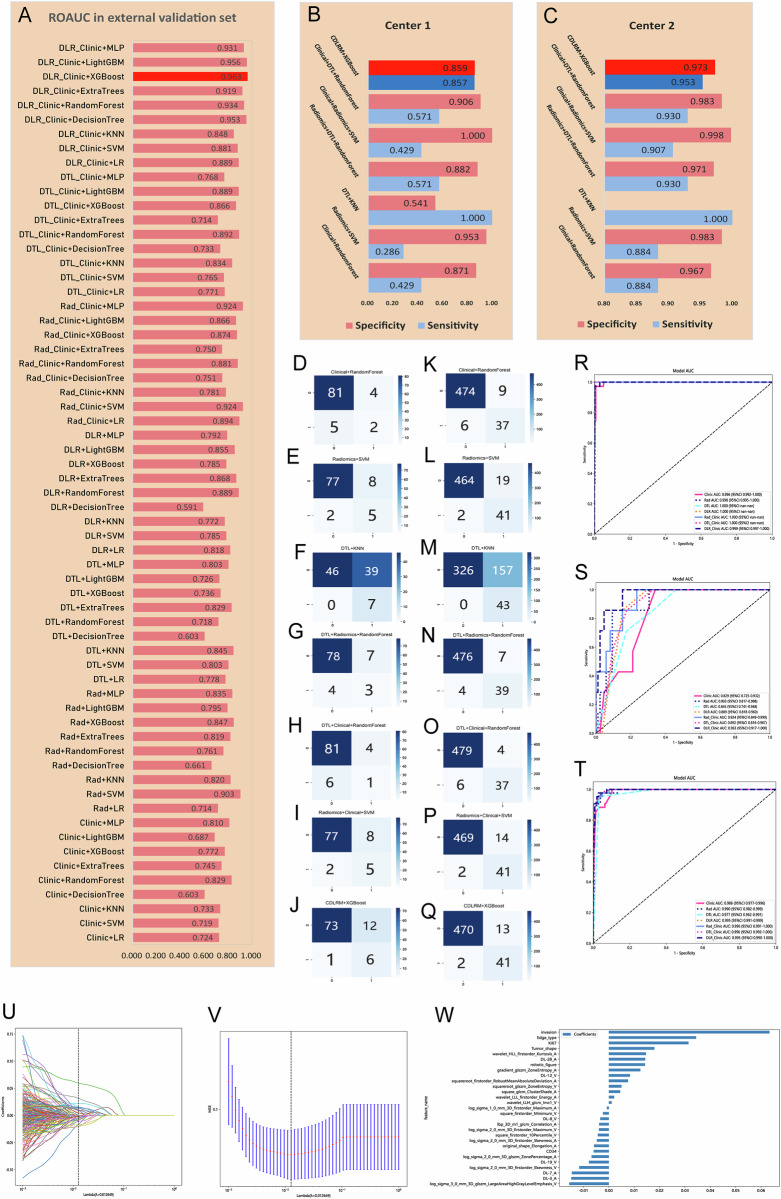
**A** Comparison of AUCs of 63 models (using machine learning combined with different feature groups), with the best values of model AUCs highlighted in red and deepened. **B**, **C** show comparison results for sensitivity and specificity across models applied to the external validation set and to the aggregation set after the initial screening. **D**–**J** show confusion matrices for different models applied to the external validation set, and **K**–**Q** show corresponding results for the aggregated set. **R**–**T** show ROCs Show ROCs for different models applied to the training set, external test, and aggregated set, respectively. **U**–**W** outline the process for constructing rad-scores for CDLRM

The extracted features were grouped into three main categories: clinical features, radiomic features, and deep learning features. These were then combined to construct seven modalities, as depicted in Fig. 3B, C. Feature selection using LASSO identified 41 features with non-zero coefficients for the logistic regression model. The final model included invasion, edge type, Ki-67, tumor shape, mitotic index, CD-34, 17 radiomic features, and 6 deep learning features. Further details are illustrated in Fig. 3W.

### Comparison of machine learning models

Nine machine learning techniques were applied to train the seven modalities, resulting in 63 models. The best-performing model for each modality was identified:

- Clinical model (random forest): AUC = 0.829 (95% CI: 0.725–0.932)
- DTL model (KNN): AUC = 0.845 (95% CI: 0.741–0.948)
- Radiomics model (SVM): AUC = 0.903 (95% CI: 0.817–0.988)
- Radiomics + DTL model (random forest): AUC = 0.889 (95% CI: 0.818–0.960)
- Clinical + DTL model (random forest): AUC = 0.892 (95% CI: 0.816–0.967)
- Clinical + Radiomics model (SVM): AUC = 0.924 (95% CI: 0.845–0.999)
- CDLRM (XGBoost): AUC = 0.963 (95% CI: 0.917–1.00)

The CDLRM model outperformed all others (Fig. 3A). Supplementary Information 9 provides ROC curves for all models tested with the internal validation set.

**Fig. 4:**
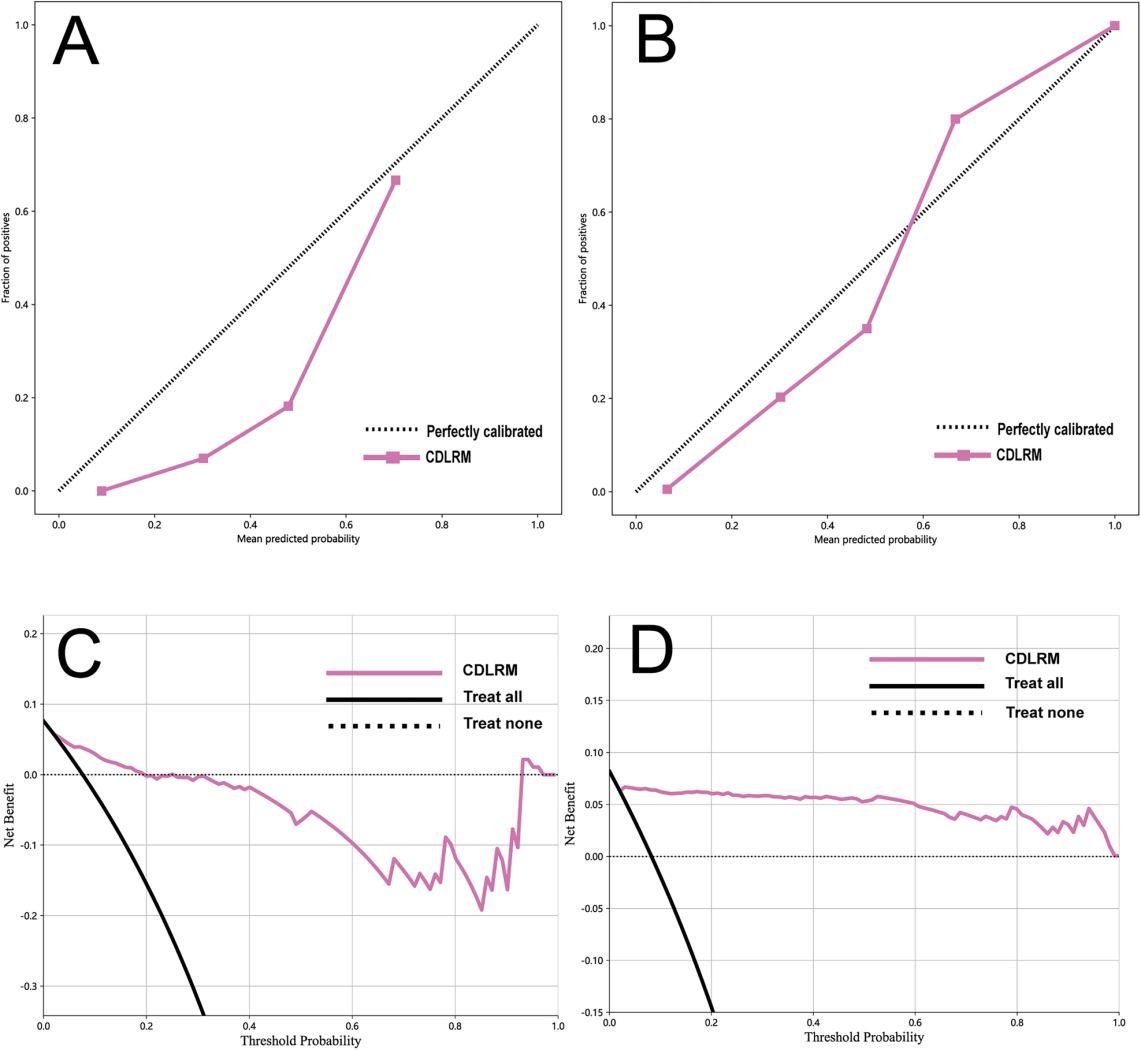
**A**, **B** show calibration curves for CDLRM applied to the external validation set and to the aggregated set, respectively. **C**, **D** show results from DCA for CDLRM applied to the external validation set and to the aggregated set

In comparing predictive performance, the CDLRM exhibited superior specificity and sensitivity. In center 1, the specificity and sensitivity were 0.859 and 0.857, respectively, while in center 2, these values were 0.973 and 0.953. In the external validation set, CDLRM identified 6 out of 7 RM cases, missing only one. Across the entire dataset, CDLRM successfully predicted 41 of 43 RM cases, with a low missed diagnosis rate meeting clinical requirements. Delong test results confirmed CDLRM’s superiority, with significant differences observed compared to the Clinical model (*p* = 0.083) and DTL model (*p* = 0.043). Calibration curves demonstrated consistency between predicted and observed outcomes (Fig. 4).

### Subgroup analysis

**Fig. 5:**
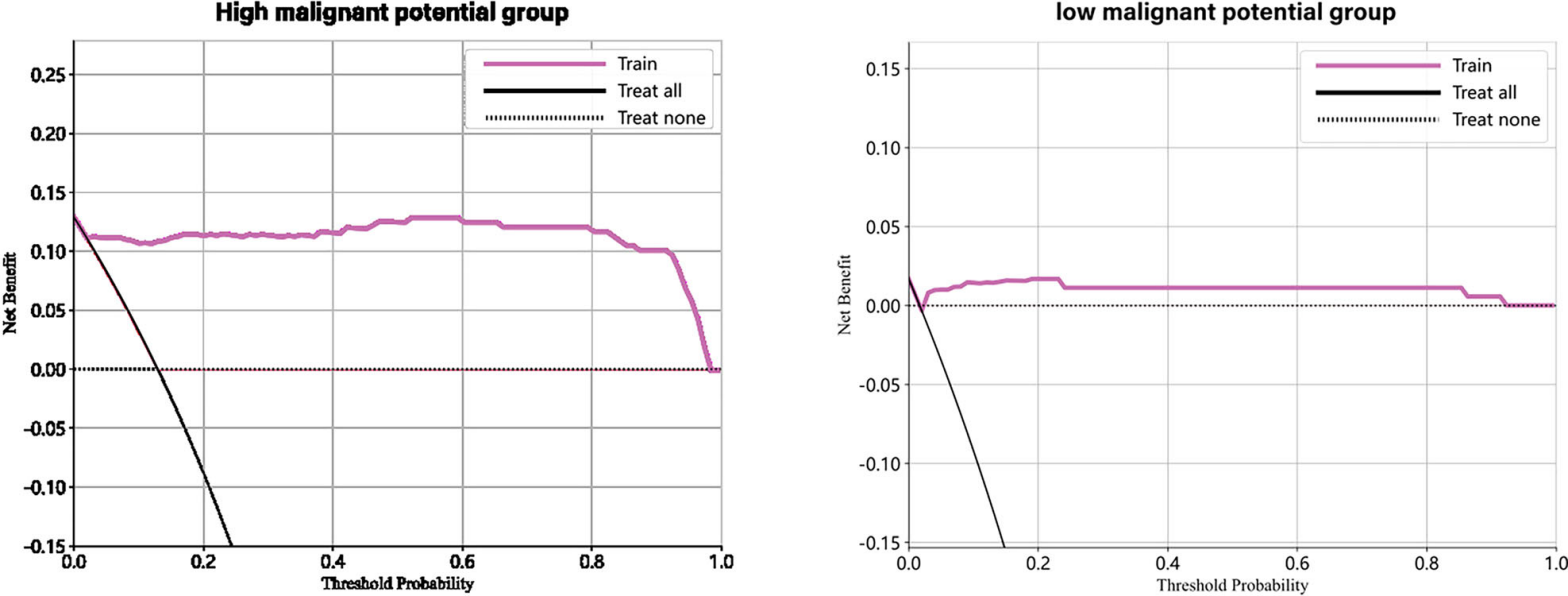
The analysis of the DCA of the high malignant potential group compared to the low malignant potential group predicted that the intervention enabled high malignant potential patients to benefit clinically to a large extent, with a high net benefit, clarifying that the clinical application scenario of CDLRM is more suitable for the high malignant potential population

Patients were categorized into two subgroups based on postoperative pathology: low malignant potential (*n* = 285) and high malignant potential (*n* = 241). CDLRM predictions were analyzed separately for each subgroup. DCA revealed a significant net clinical benefit in the high malignant potential group, emphasizing the model’s utility in guiding follow-up and intervention strategies. In contrast, the low malignant potential group showed limited clinical benefit, with intervention strategies providing negligible advantage (Fig. 5).

## Discussion

The widespread use of imatinib has significantly reduced the incidence of recurrence and metastasis (RM) in patients after surgery. For instance, in our study, while 198 GIST patients were pathologically diagnosed as high-risk, only 43 developed RM. Considerable effort has been made to assess the risk of recurrence following R0 resection for GISTs, with widely accepted methods such as the modified NIH criteria, Armed Forces Institute of Pathology risk stratification, and prognostic contour maps [8, 19, 20]. These approaches are largely equivalent in their effectiveness. The landmark work by Heikki Joensuu et al on the risk of recurrence after surgery for GISTs solidified the modified NIH risk stratification as a cornerstone of precision medicine in GIST treatment. However, the risk of RM in patients receiving adjuvant imatinib has been understudied, largely due to the difficulty of assessing its benefit [21].

With advancements in clinical practice, deep learning and radiomics have emerged as promising tools [22]. Our study combines these techniques to extract features from multiple perspectives, using machine learning methods to develop the CDLRM model aimed at predicting RM in postoperative patients on oral imatinib. The predictive performance of this model has been highly satisfactory.

### Key features in CDLRM

The CDLRM model incorporated features such as Ki-67, type of surgery, edge type, tumor shape, growth pattern, invasion, intratumoral degeneration, CT enhancement mode, modified NIH risk stratification, and mitotic index, which were all significantly associated with RM. These findings align with prior research. Interestingly, the study by Guy J. C. Burkill et al highlighted a correlation between tumor maximum diameter and malignant potential, but our findings did not confirm this. Among the 36 RM cases at Center 1, 14 had a maximum diameter ≤ 5 cm, and at Center 2, three of the seven RM cases had a similar size. This suggests that even small GISTs can recur or metastasize. The absence of statistical significance for tumor size in our study may reflect advances in surgical techniques and postoperative imatinib use, which allow prolonged RM-free intervals regardless of tumor size.

In Center 2, the type of surgery was not significantly associated with RM, possibly due to a higher prevalence of open surgery at the time of patient inclusion. Notably, none of the patients who underwent laparoscopic surgery experienced RM, which could skew the statistical results. Additionally, tumor shape and CT enhancement mode were not statistically significant in Center 2, likely due to the smaller sample size. Future studies with larger external validation sets may address these limitations.

### Feature weighting analysis

In the feature weighting analysis, invasion, Ki-67, tumor shape, edge type, mitotic index, two deep learning features, and seven radiomic features showed positive correlations with RM. Among these, invasion had the highest weighting, indicating its critical role in RM. Invasive tumors often leave residual tissue post-surgery, leading to RM. Ki-67, a marker of tumor proliferation, also showed a strong correlation with RM, consistent with previous findings [23].

Radiomic features such as WaveletHLL-firstorder-Kurtosis, Squareroot-firstorder Robustmeanabsolute deviation, and WaveletLLL-firstorder-Energy were positively correlated with RM. These features reflect heterogeneity within the tumor, with higher values indicating greater potential for RM. Metrics like ZoneEntropy and cluster shade further highlight textural irregularities as significant factors influencing RM [24].

### Model comparison and clinical implications

**Fig. 6:**
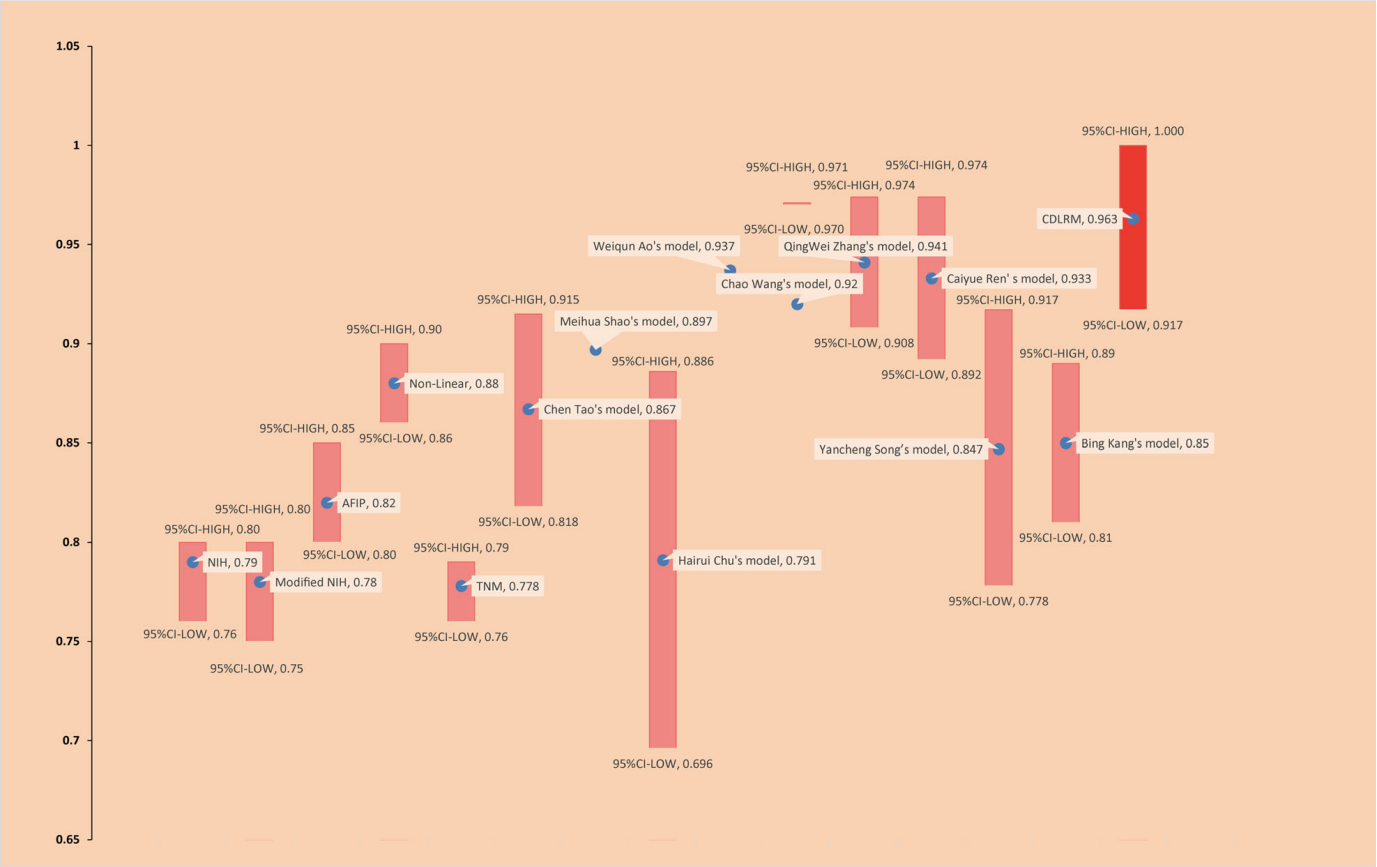
CDLRM is compared with models adopted by previous studies. For some models, we do not include the 95% CI because we could not obtain this information from the original article. The only single-center investigation is by Chu and collaborators

Cross-sectional comparisons with 14 previous models confirmed that CDLRM outperformed other approaches, achieving superior AUCs on the external validation set (Fig. 6). For further comparison, in terms of cohort selection, our study with Chen Tao [10], ChingWei Zhang [25], Yancheng Song [26], and Bing Kang et al [27] chose a multicentre cohort. External cohort validation was also used to generalize the predictive performance of the model. While most prior studies combined radiomic and clinical features, only Bing Kang et al and our study incorporated deep learning features into model construction. Unlike Bing Kang et al, who focused solely on deep learning, we employed classic machine learning for model training, ensuring high repeatability and broader applicability [9, 28–32].

CDLRM allows clinicians to optimize follow-up strategies for GIST patients, particularly those at high risk of RM, thereby improving prognoses and promoting precision medicine. To explore its clinical applications, we propose dividing patients into subgroups: those with high malignant potential requiring imatinib and those with low malignant potential requiring observation. DCA curve analysis revealed that CDLRM provided substantial clinical benefits for the high-malignant-potential subgroup by facilitating early detection and intervention.

### Limitations and future directions

While the use of SMOTE oversampling and five-fold cross-validation minimized overfitting and improved model accuracy, certain limitations remain. For example, our study did not account for genetic mutations due to the lack of a sufficiently large cohort with c-Kit or PDGFRA test results [33]. Future research should explore the prognostic impact of genetic mutations on RM and validate CDLRM through prospective studies.

Variability in CT equipment, ROI delineation methods, and software inconsistencies are additional challenges to the reproducibility and clinical applicability of radiomics research. Establishing consensus guidelines for these factors will be essential for future progress in this field.

## Conclusion

The CDLRM model demonstrated excellent performance in predicting RM in primary GISTs, especially among high-malignant-potential patients. By guiding clinical decisions and optimizing personalized treatment plans, this model represents a significant step toward advancing precision medicine in GIST management.

## Supplementary information

ELECTRONIC SUPPLEMENTARY MATERIAL

## Data Availability

The datasets involved in this study are still under further research and are not publicly available at this time. However, they can be obtained from the corresponding authors upon reasonable request.

## Abbreviations

CDLRM: Clinical deep learning radiomics model
CNN: Convolutional neural network
DCA: Decision curve analysis
DTL: Deep transfer learning
GISTs: Gastrointestinal stromal tumors
LASSO: Least absolute shrinkage and selection operator
NIH: National Institutes of Health
NRM: Non-recurrence or metastasis
ROI: Regions of interest
RM: Recurrence or metastasis
SVM: Support vector machine
XGBoost: eXtreme gradient boosting
