# Supplementary material for: A machine learning model integrating clinical-radiomics-deep learning features accurately predicts postoperative recurrence and metastasis of primary gastrointestinal stromal tumors
Source: Insights Imaging. 2025 Jun 26;16:135. doi: 10.1186/s13244-025-02011-8 (PMC12202268; doi:10.1186/s13244-025-02011-8)

A machine learning model integrating Clinical-Radiomics-Deep learning features accurately predicts postoperative recurrence and metastasis of primary gastrointestinal stromal tumors

ELECTRONIC SUPPLEMENTARY MATERIAL

Supplementary Information 1:
Detailed information on the definitions of the relevant terms.

Definitions of relevant terms are referenced from the National Comprehensive Cancer Network (NCCN) guidelines. We adopted the following definition of RM: progression (appearance of a new lesion, recurrence at the primary site, or liver metastasis to other sites) after primary GISTs in patients who underwent R0 surgery, confirmed by pathological puncture results or by joint review of CT results by two physicians. R0 resection: This refers to the absence of cancer cells in the cut line under the pathological section. The result was obtained from the patient's postoperative pathology report. High malignant potential: means a risk classification of medium or high risk under the modified NIH criteria. Low malignant potential: refers to a risk classification of very low risk or low risk under the modified NIH criteria. Our follow-up criteria involved CT of the abdomen or pelvis every 3–6 months for patients at intermediate or high risk of complete resection. For low-risk or small GISTs (less than 2 cm), CT was performed once a year. We defined outcomes as recurrence or metastasis (RM) identified during postoperative follow-up. Non-RM (NRM) was defined as no RM (no progression) in patients monitored over a period of 3 or more years.

GIST patients are required to take oral imatinib according to National Comprehensive Cancer Network (NCCN) guidelines, with low-risk and extremely low-risk drugs not taken orally, and moderate to high-risk oral imatinib drugs.

Supplementary Information 2: Detailed definition information for CT features.

margin contour (Clear: tumor contour is sharp-like and clearly distinguishable. Blurred: tumor contour is indistinguishable); Tumor shape(Round: overall margin is soft and round or round-like. Lobulated: Multiple round or round-like protrusions, originating from one base. Irregular: tumor shape cannot be described.); Tumor growth pattern (Internal: growth toward the inside of the digestive tract. External: growth toward the outside of the digestive tract. Mixed: the direction of growth cannot be determined); Tumor invasion(the relationship between tumor and adjacent tissues is considered non-invasive if the gap is clear, and invasive if the gap is blurred); Tumor enhancement mode(Uniform enhancement, Non-uniform enhancement. According to CT written report); Intratumoral degeneration(Yes: necrotic liquefaction foci were observed. No: not observed.) Tumor calcification (Yes: high-density calcified foci were observed. No: not observed).

Supplementary Information 3: CT scanning protocol and related parameter content.

CT protocol

Patients underwent CT examinations performed using two CT scanners (Phillips 256 I CT, Philips Health-care System; Revolution CT, GE Healthcare). Patients abstained from drinking liquids for more than 6 hours before CT examination, and consumed approximately 600–1000 ml of water within 15 minutes before CT scanning. The CT examinations, which covered the whole abdomen and pelvis, were performed during breath-hold with the patient in a head-up supine position. After routine unenhanced CT was performed, patients received an infusion of nonionic contrast agent (iohexol, 1.5 mL/kg administered at 3.0 mL/s, 350 mg I/mL; Iopamidol Injection Bracco) into the antecubital vein, followed by a 20-mL saline flush. CT series for arterial phase (AP) and venous phase (VP) were obtained after post-injection delays of 30 and 70 seconds, respectively. Acquisition parameters were: tube voltage, 120 kVp; tube current, 120–550 mA; rotation time, 0.5 seconds; FOV, 500 × 500 mm; matrix size, 512×512. Images were reconstructed with a standard kernel. Reconstructed section thickness was 5 mm.

Supplementary Information 4:
the detailed process of radiomics features as well as deep transfer learning feature processing was described.

1. Radiomic features

   1.1 Pre-processing

Considering that CT images come from different centers, different scanners or different scanning protocols, their voxel spacing is heterogeneous. We performed the following preprocessing steps:

(1) Hide patient information;
(2) Pixel normalization: pixel values within each image are sorted, and the intensity is truncated to a range between 0.5 and 99.5%;
(3) Image resampling at fixed resolution;
(4) Data labeling.

   1.2 ROI segmentation and feature extraction

Four physicians participated in tumor segmentation. The drawing tool used was 3D-Slicer (open-source version 4.13.0 http://www.slicer.org/). We extracted hand-labeled features using Pyradiomics (http://pyradiomics.readthedocs.io). A previous study showed that there was no significant difference between 2D and 3D feature assessments[1]. To optimize the process and improve reproducibility, we selected the largest tumor slice within the 3D image for ROI definition. On the basis of inspection of 60 patients randomly selected from the training set for intraclass correlation coefficient (ICC) analysis, we regarded features with ICC≥0.90 to be robust against intra-rater and inter-rater uncertainties.

   1.3 Feature filtering

We performed Mann-Whitney U-tests on data from radiomic features, and retained

features associated with P<0.05. The correlation between features was then calculated using Spearman correlation coefficients, and retained one of the features with a correlation coefficient greater than 0.9 between any two features. We then performed feature filtering using a greedy recursive deletion strategy, removing features with the greatest redundancy in the current set one at a time. Least absolute shrinkage and selection operator (LASSO) regression was used for the final filtering. We performed 10-fold cross-validation to minimize feature errors associated with the preserved non-zero coefficients. Finally, we obtained radiomic scores for each patient via linear combination of the retained features, weighted by the model coefficients. The code implementing feature filtering is publicly available. (https://github.com/Shegeweiwu/xiewenjiecode.git). Our entire codebase was developed using the OnekeyAI platform. The code we have made open source can be executed on an authorized Onekey platform.

2. Features from deep transfer learning

2.1 Pre-processing

The ROI region with the largest cross-sectional area was cropped, and the grayscale values were normalized to the [-1,1] range using the min-max transform. Next, each cropped subregion was resized to 224×224 via nearest interpolation. We resized all cropped data to 224x224 pixels and employed horizontal and vertical flipping as our data augmentation techniques to prevent overfitting during the training process of the model. The specific details of this optimiser were as follows:

We carefully set the learning rate for better generalization and to mitigate leak of image data. In this study, we adapted the cosine decay learning rate algorithm. Our learning rate is specified by the following expression:

$$\eta_t^{task-spec} = \eta_{min}^i + \frac{1}{2}\left(\eta_{max}^i - \eta_{min}^i\right)\left(1 + cos\left(\frac{T_{cur}}{T_i}\pi\right)\right)$$

The parameters $\eta_{min}^i = 0$, η_{max}^i *=0.01, and* $T_i = 30$ *represents the minimum learning rate, the maximum learning rate, and the number of iteration epochs, respectively. Because the backbone part adopts pre-training parameters in order to ensure the migration effect,* $T_{cur} = \frac{1}{2}T_i$ *to fine-tune the parameters associated with the backbone part. Therefore, the learning rate of the backbone part is:*

$$\eta_t^{backbone} = \begin{cases} 0 & \text{if } T_{cur} \le \frac{1}{2}T_i \\ \eta_{min}^i + \frac{1}{2}\left(\eta_{max}^i - \eta_{min}^i\right)\left(1 + cos\left(\frac{T_{cur}}{T_i}\pi\right)\right) & \text{if } T_{cur} > \frac{1}{2}T_i \end{cases}$$

We used stochastic gradient descent as optimizer, and sigmoid cross-entropy as loss function.

2.2 Feature extraction and compression

We extracted features from a pre-trained CNN via deep transfer learning (DTL). We selected Resnet50 trained on the ImageNet ILSVRC-2012 dataset as the pre-trained CNN model. Because the native dimension of the DTL features was 2048, we used PCA to reduce it to 128 dimensions. This procedure ensures good balance across features, and reduces the risk of overfitting. We then standardized all compressed features via Z-scoring, and calculated mean and variance of each column of features. Each column of features was subtracted from the mean, divided by the variance, and transformed into a standard normal distribution. We used the same method to filter the DTL feature set. To investigate the interpretability of DTL, the network was visualized by applying gradient-weighted class activation mapping (Grad-CAM) to generate a coarse localization map for GISTs. We found that the localization map matched the actual location of the tumor (As shown in the figure below). This result confirms that the origin of the DTL feature was directly associated with the tumor, making the model more convincing.

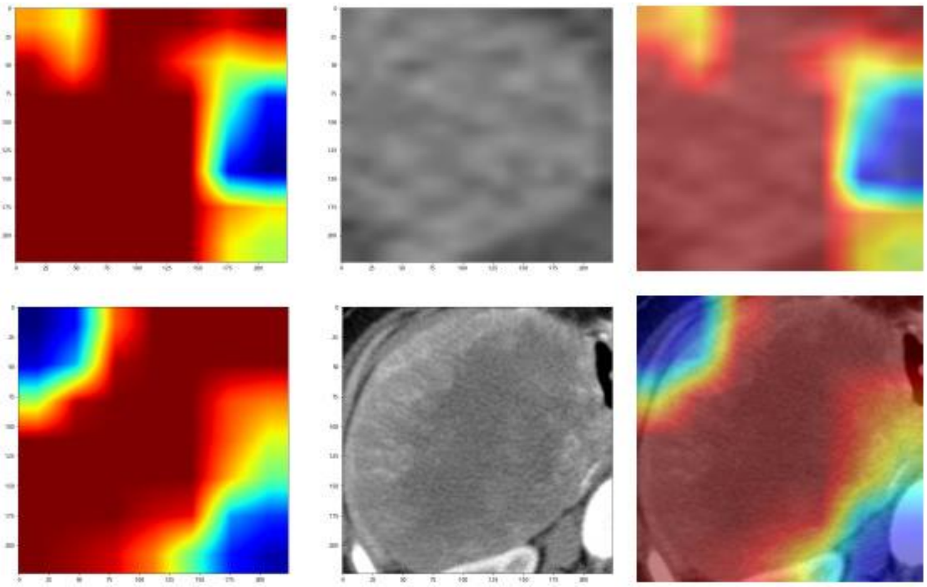

Tumour segmentation and corresponding heat map. Red and yellow regions indicate target regions activated by DTL with the highest predictive significance; blue background reflects regions with weaker predictive values.

1. Lubner, M.G., et al., *CT textural analysis of hepatic metastatic colorectal cancer: pre-treatment tumor heterogeneity correlates with pathology and clinical outcomes.* Abdom Imaging, 2015. **40**(7): p. 2331-7.

Supplementary Information 5:

Details of algorithms and parameters relating to extraction and modelling methods for radiomics features and deep learning features.

- normalize_method: The normalization method used, specifically 'imagenet' in this case.
- model_name: The name of the model used, specifically 'resnet50'.
- gpus: The GPUs utilized for the computations, with GPU 0 being selected.
- batch_size: The batch size used during training and evaluation, set to 32.
- epochs: The number of epochs the model was trained for, with a value of 30.
- init_lr: The initial learning rate for the optimization algorithm, set to 0.01.
- optimizer: The optimization algorithm used, specifically 'sgd' (Stochastic Gradient Descent).

These parameters provide an overview of the experimental setup for the described model and its training process.

Supplementary Information 6:

Follow-up information for relapsed patients, with patient names hidden.

Name	Date of diagnosis	Follow-up date	Follow-up period/Month
N1	2016/11	2021/12	61.87
N2	2016/12	2021/12	60.90
N3	2016/10	2021/12	62.97
N4	2016/3	2021/12	70.13
N5	2016/11	2021/12	62.00
N6	2017/3	2021/12	58.03
N7	2017/9	2021/12	51.93
N8	2018/7	2021/12	41.90
N9	2017/7	2021/12	54.10
N10	2019/2	2021/12	34.80
N11	2019/11	2022/4	29.77
N12	2019/5	2021/12	31.90
N13	2016/10	2021/12	63.33
N14	2019/1	2021/12	35.97
N15	2018/11	2021/12	38.03
N16	2019/9	2021/12	27.93
N17	2019/3	2021/12	34.10
N18	2019/9	2022/3	31.00
N19	2019/5	2021/12	32.13
N20	2019/7	2021/12	30.13
N21	2019/5	2021/12	32.20
N22	2019/10	2022/3	30.13
N23	2019/3	2021/12	34.30
N24	2015/9	2021/12	76.90
N25	2015/4	2021/12	82.03
N26	2015/4	2021/12	82.07
N27	2015/7	2021/12	79.07
N28	2016/5	2021/12	68.93
N29	2016/8	2021/12	65.90
N30	2016/11	2021/12	62.87
N31	2017/7	2022/1	53.83
N32	2018/1	2022/1	48.47
N33	2015/4	2022/1	82.10
N34	2017/9	2022/1	52.70
N35	2019/11	2022/5	30.30
N36	2015/12	2022/1	74.13
N37	2017/12	2022/1	49.13
N38	2018/3	2022/1	46.70
N39	2018/3	2022/1	46.53
N40	2019/5	2022/1	32.37
N41	2015/12	2022/1	74.07
N42	2019/5	2022/1	32.27
N43	2019/4	2022/1	33.07
Follow-up time 1-3 years=15			
Follow-up time 3-5 years=12			
Follow-up time ＞5 years=16			

Supplementary Information 7:
Corresponding p-values for all extracted radiomics features.

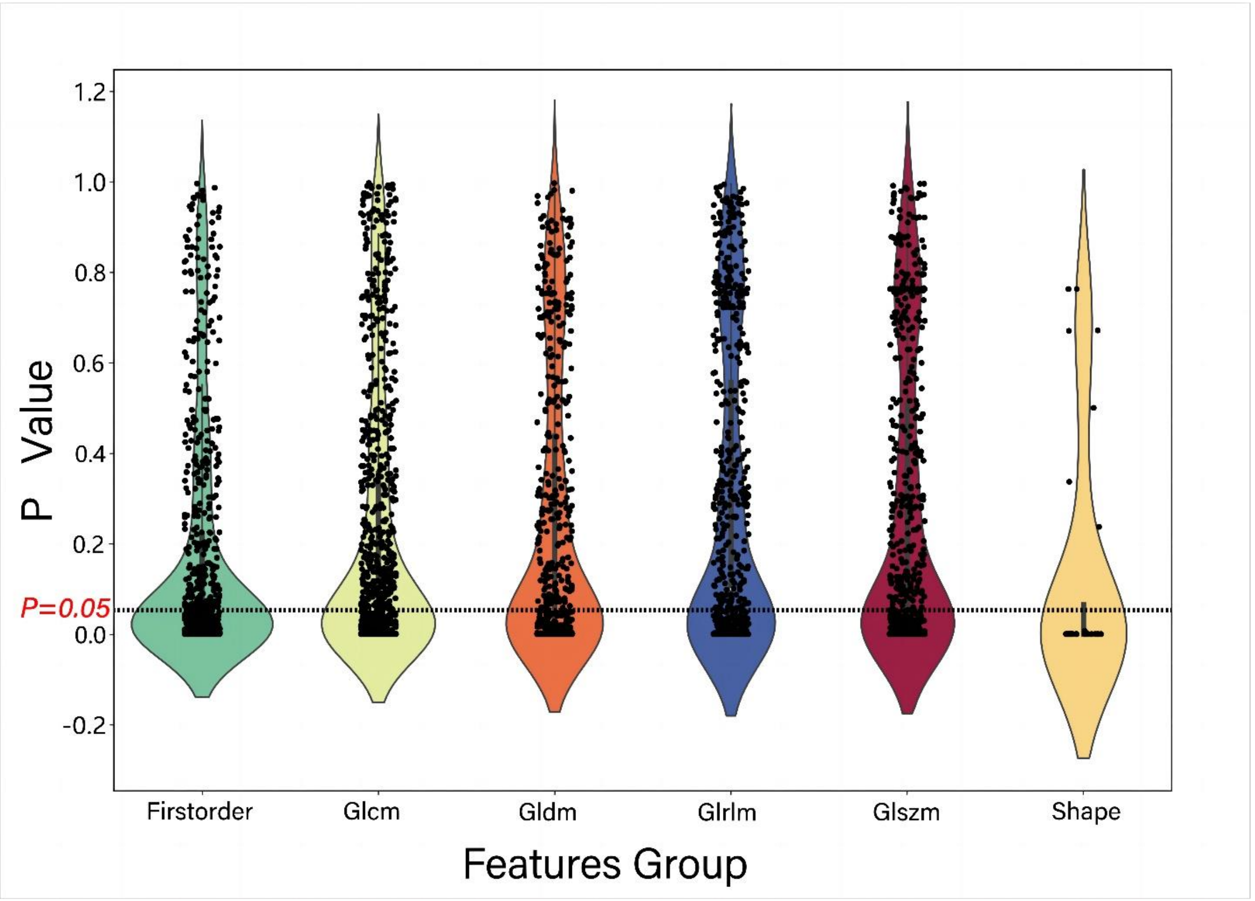

Supplementary Information 8: Correlation coefficient of each deep transfer learning features.

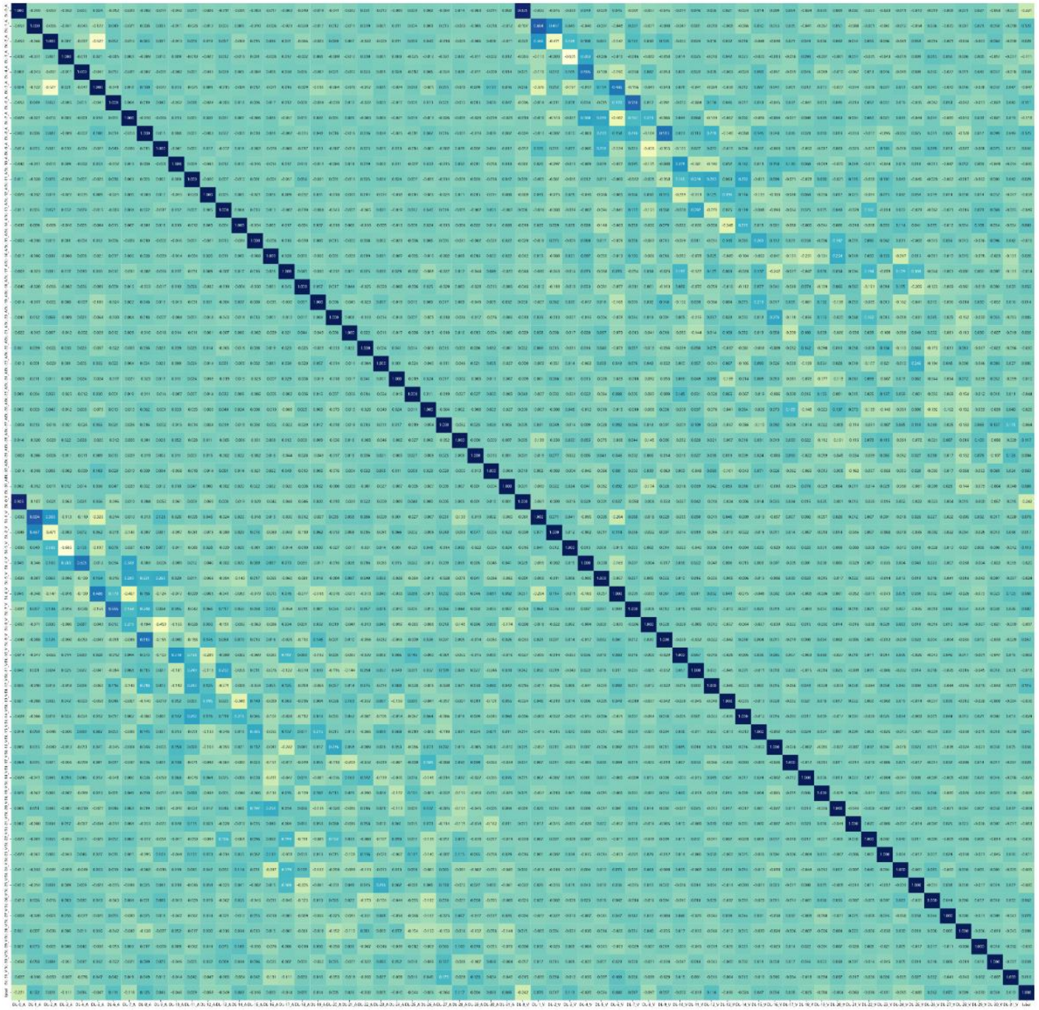

Supplementary Information 9:
This figure shows the predictive performance, i.e., AUC values, of nine machine learning models in the internal validation set under seven modals.

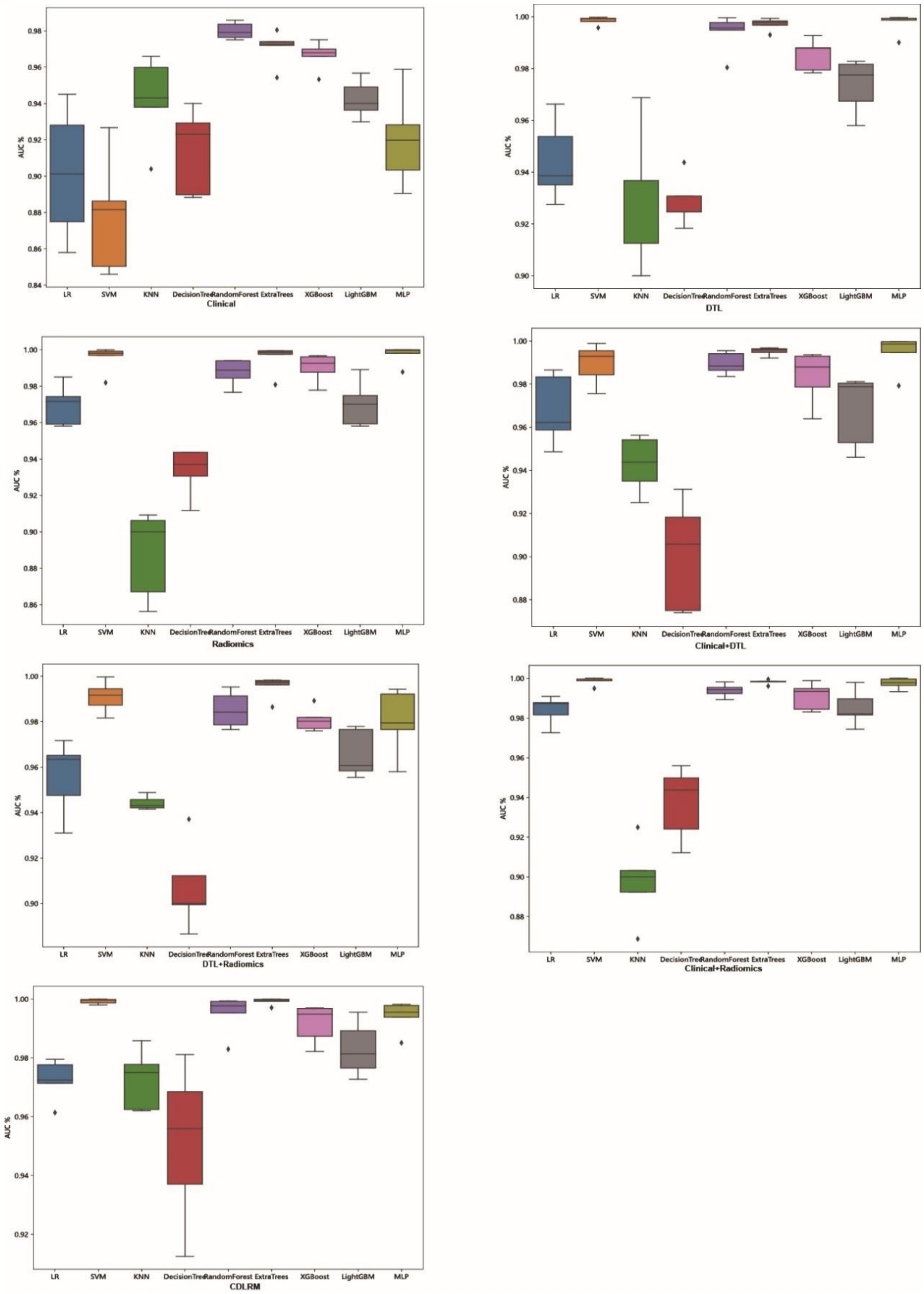

Supplement: Supplementary file 1 — ELECTRONIC SUPPLEMENTARY MATERIAL [file 13244_2025_2011_MOESM1_ESM.pdf]
